# Association between red cell distribution width and mortality in patients undergoing continuous ambulatory peritoneal dialysis

**DOI:** 10.1038/srep45632

**Published:** 2017-04-03

**Authors:** Yao-Peng Hsieh, Shr-Mei Tsai, Chia-Chu Chang, Chew-Teng Kor, Chi-Chen Lin

**Affiliations:** 1 Division of Nephrology, Department of Internal Medicine, Changhua Christian Hospital, Changhua, Taiwan; 2 Ph.D. program in translational medicine, College of Life Science, National Chung Hsing University, Taichung, Taiwan; 3 School of Medicine, Kaohsiung Medical University, Kaohsiung, Taiwan; 4 School of Medicine, Chung Shan Medical University, Taichung, Taiwan; 5 Department of Nursing, Changhua Christian Hospital, Changhua, Taiwan; 6 Institute of Biomedical Sciences, College of Life Science, National Chung Hsing University, Taichung, Taiwan

## Abstract

Although red cell distribution width (RDW) has emerged as a biomarker of clinical prognostic value across a variety of clinical settings in the last two decades, limited evidence is available for its role in end-stage renal disease. We enrolled 313 incident patients undergoing continuous ambulatory peritoneal dialysis (CAPD) in this retrospective observational study from 2006 to 2015. In the fully adjusted model of Cox regression analysis, the adjusted hazard ratios for the high RDW group versus the low RDW group were 2.58 (95% confidence interval (CI) = 1.31–5.09, p = 0.006) and 3.48 (95% CI = 1.44–8.34, p = 0.006) for all-cause and cardiovascular disease (CVD)-related mortality, respectively. Based on area under the receiver operating characteristic curve (AUC) analysis, RDW (AUC = 0.699) had a stronger predictive value for all-cause and CVD-related mortality than other biological markers including hemoglobin (AUC = 0.51), ferritin (AUC = 0.584), iron saturation (AUC = 0.535), albumin (AUC = 0.683) and white blood cell count (AUC = 0.588). Given that RDW is a readily available hematological parameter without the need for additional cost, we suggest that it can be used as a valuable index to stratify the risk of mortality beyond a diagnosis of anemia.

The health burden of chronic kidney disease (CKD) is increasing globally, especially for those with advanced CKD. Peritoneal dialysis (PD) is one of the accepted renal replacement therapies for patients reaching end-stage renal disease (ESRD). A total of 27,522 ESRD patients were treated with PD in 2009 according to the 2011 US Renal Data System report^1^. Despite an increased understanding of pathophysiological processes in patients undergoing PD and subsequent improvements in management strategies, the mortality rate associated with PD is still high. The leading cause of mortality is cardiovascular disease (CVD), accounting for 40–50% of all deaths^2^.

Anemia is prevalent in CKD patients, mainly because of decreased erythropoietin production by dysfunctional kidneys, and this has also been associated with CVD-related mortality in patients undergoing PD^3^. Red cell distribution width (RDW), an index of quantitative measurements of erythrocyte volume variability, is routinely reported as part of a complete blood cell count (CBC). It is calculated by dividing the standard deviation of the mean corpuscular volume (MCV) by the MCV of erythrocytes. Traditionally, RDW is used along with MCV for the differential diagnosis of anemia, especially in iron deficiency anemia^4^. However, it has also emerged as a biomarker of clinical prognostic value across a variety of clinical settings in the last two decades^5^,^6^,^7^,^8^,^9^. An elevated RDW level, even in the reference range, has also been associated with an increased risk of mortality^10^,^11^,^12^.

The exact mechanism explaining the association between RDW and mortality is unknown. In CKD patients, RDW has been reported to be closely associated with renal function status^13^. However, limited evidence is available for the predictive role of RDW in the risk of mortality in patients with CKD, particularly those treated with PD. Therefore, the aim of this retrospective investigation was to investigate the association between RDW and the risk of both all-cause and CVD-related mortality in patients undergoing continuous ambulatory peritoneal dialysis (CAPD) between 2006 and 2015 in a medical center in Taiwan.

## Results

### Patient characteristics

The study cohort included 313 patients undergoing CAPD from 2006 to 2015. The baseline characteristics of these patients stratified by median RDW value (15.3%) are shown in Table 1. The mean age was 54.5 ± 15.9 years, and 164 (52.4%) were male. The three leading causes of ESRD were chronic glomerulonephritis (34.1%), diabetes mellitus (28.1%) and hypertension (17.8%). Most of the patients (248, 79.2%) had pre-dialysis CKD before initiating PD. At baseline, the patients in the higher RDW group (>15.3%) were older, had lower urine output and lower residual renal function. With regards to laboratory examinations, the patients in the high RDW group had lower levels of albumin, calcium, hemoglobin, and cholesterol, and higher levels of alkaline phosphate, ferritin and blood urea nitrogen (BUN) compared to the lower RDW group (≦15.3%). With regards to pharmacotherapy, more patients in the low RDW group used iron preparations.

### Association of RDW with all-cause and CVD-related mortality

**Figure 1:**
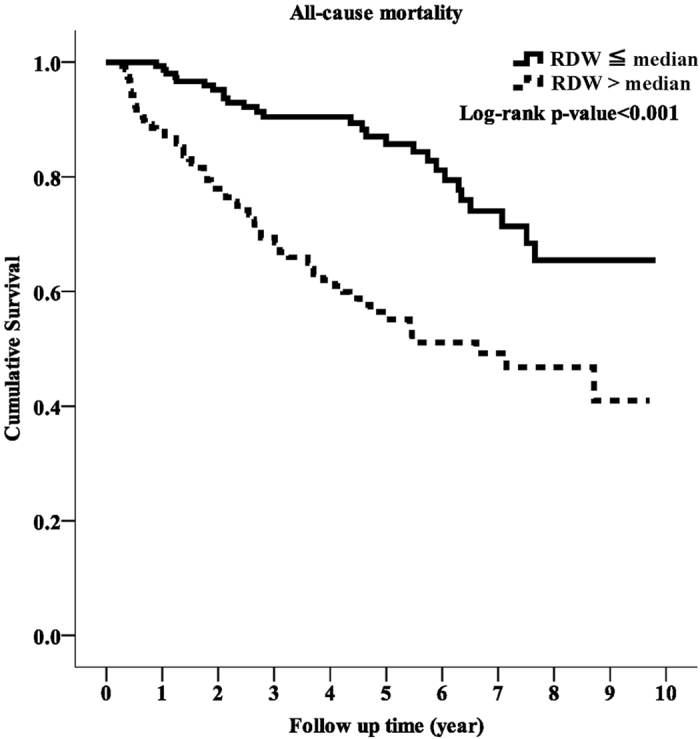
Kaplan-Meier curve of overall patient survival according to the RDW groups (log-rank test, p < 0.001).

**Figure 2:**
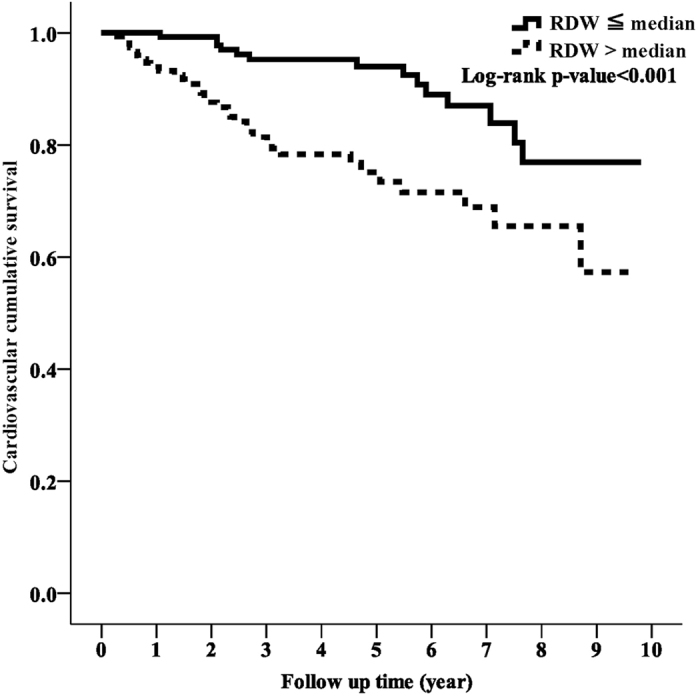
Kaplan-Meier curve of cumulative survival free of cardiovascular disease-related mortality according to the RDW groups (log-rank test, p < 0.001).

**Figure 3:**
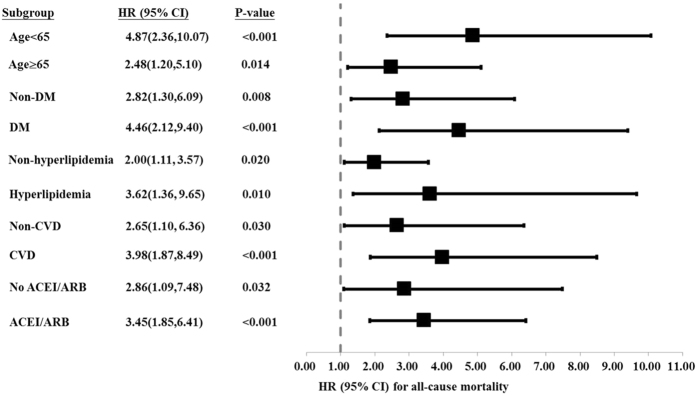
Subgroup analysis showing adjusted hazard ratios for all-cause mortality in the high RDW group compared with the low RDW group.

**Figure 4:**
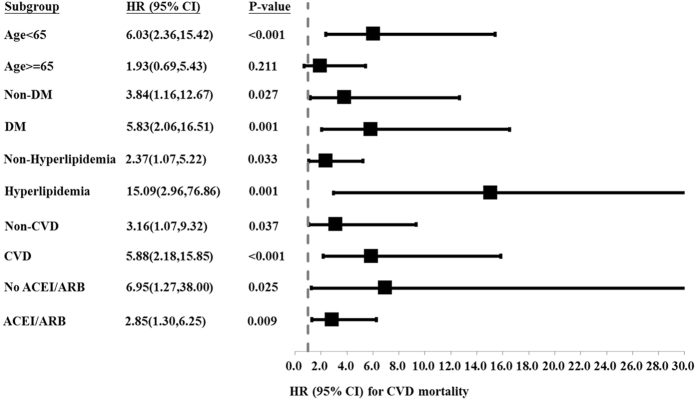
Subgroup analysis showing adjusted hazard ratios for cardiovascular disease-related mortality in the high RDW group compared with the low RDW group.

During the study period, 27 patients (17.4%) died in the low RDW group and 64 patients (40.5%) died in the high RDW group (p < 0.001). Of these 91 patients, 48 died of CV events, which was the leading cause of mortality. There was also a significant difference in CVD-related mortality rate between the two groups, with 14 patients (9%) in the low RDW group and 34 (21.5%) in the high RDW group (p = 0.003). Kaplan-Meier survival curves showed that the high RDW group had higher all-cause and CVD-related mortality rates compared to the low RDW group (Figs 1 and 2; p < 0.001, p < 0.001, respectively). In the unadjusted and adjusted Cox proportional regression models, the high RDW group was associated with an increased risk of all-cause and CVD-related mortality compared with the low RDW group (Table 2). In the fully adjusted model (model 5), the adjusted HRs for the high RDW group versus the low RDW group were 2.58 (95% CI = 1.31–5.09, p = 0.006) and 3.48 (95% CI = 1.44–8.34, p = 0.006) for all-cause and CVD-related mortality, respectively. Subgroup analyses showed that the patients with higher RDW levels had higher rates of all-cause and CVD-related mortality in the adjusted models compared to those with lower RDW levels (Figs 3 and 4).

### Sensitivity analysis

Three levels of sensitivity testing were performed as shown in Table 2. In the fully adjusted model, a higher RDW level was associated with a higher risk of overall and CVD-related mortality in all of the three sensitivity analyses.

### Association of RDW with different variables

Table 3 shows the strength of association and correlation of RDW with other parameters by Pearson correlation test and linear regression analysis. A negative association was disclosed between RDW and albumin, hemoglobin, intact parathyroid hormone, body mass index, and cholesterol. However, these associations were weak (the absolute value of Pearson correlation < 0.3). Albumin had the strongest correlation with RDW, with a 0.64% decrease with every 10-g/L increase in albumin level (Pearson coefficient, −0.196).

### Predictive value of RDW

We first calculated the area under the curve (AUC) in receiver operating characteristic (ROC) analysis to compare the predictive value of a single variable in predicting overall and CVD-related mortality within 1-year, 3-year and 5-year periods. As shown in Table 4, RDW had the highest predictive value compared to the other variables over the study period, except for albumin which had the highest value in predicting 3-year overall mortality. We then calculated the AUC after adding each variable to model 4 (Table 4). Adding RDW to model 4 resulted in the highest AUC compared to the other variables, implying that RDW was a better index in predicting 1-year, 3-year and 5-year overall and CVD-related mortality.

## Discussion

In this study, we investigated the relationship between baseline RDW levels and patient survival in 313 incident CAPD patients over a period of 10 years at a single PD center, and found a robust and consistent relationship between high RDW and overall and CVD-related mortality independent of other common risk factors. In addition, RDW was superior to albumin, ferritin, WBC, iron saturation and hemoglobin in predicting the risk of overall and CVD-related mortality based on AUC analysis in both univariate and multivariate models. In contrast to previous studies on RDW, the association between RDW and laboratory variables was weak with a negative association with albumin being the strongest correlation.

RDW has been used to differentiate the causes of anemia in clinical practice. A high degree of heterogeneous red blood cell (RBC) size is called anisocytosis. An elevated RDW is commonly encountered in patients with impaired erythrocyte production or increased erythrocyte destruction. In recent studies, RDW has been reported to be a significant prognostic marker for the risk of mortality in various diseases, and especially cardiovascular diseases^14^. Considerable and convincing evidence has indicated the close relationship between RDW and acute coronary syndrome, ischemic cerebrovascular disease, peripheral artery disease, atrial fibrillation and heart failure^14^. A high RDW level has also been reported to predict adverse outcomes in patients with these conditions. A meta-analysis of 17 cohort studies by Huang *et al*. showed the prognostic role of RDW on admission and discharge in patients with congestive heart failure with a 10% increase in the overall risk of mortality for every 1% increase in baseline RDW^15^. Furthermore, in a Chinese population of 1,442 patients with stable angina, a higher RDW on admission was shown to increase the risk of 1-year cardiac mortality and 1- year acute coronary syndrome^16^.

In addition to the prediction of a higher risk of mortality in patients with cardiovascular diseases, RDW has also been implicated in the clinical setting of kidney diseases. Oh *et al*. reported that RDW at the initiation of continuous renal replacement therapy was an independent predictor of 28-day all-cause mortality after multiple adjustments^17^. Later, a single-center, prospective longitudinal study of 100 hemodialysis patients reported that a RDW value above the median was associated with a hazard ratio of 5.15 for 1-year mortality compared to a lower RDW value^18^. More recently, a large retrospective observational cohort study of 109,673 adult patients undergoing maintenance hemodialysis concluded that a higher RDW was strongly associated with a higher risk of mortality, and that it was also a much stronger predictor of mortality compared to traditional makers of anemia^19^. Furthermore, Peng *et al*. conducted a single-center study of 1,293 incident PD patients, and found that the higher RDW group had a 60% higher risk of CV-related mortality compared to the lower RDW group, although the association between RDW and all-cause mortality did not reach statistical significance in the fully adjusted model^20^. A recent investigation of PD patients in Korea was conducted by Sun *et al*. who showed that RDW levels at PD initiation were associated with all-cause mortality, but not with fatal cardiovascular events^21^. In the current study, we found a significant correlation between RDW and both the risk of CVD-related mortality and all-cause mortality. The difference in results between our study and Peng *et al*. and Sun *et al*. may partly be due to distinct patient characteristics and diverse healthcare delivery.

Although the exact pathophysiological mechanisms are unknown, substantial evidence suggests a robust and independent relationship between RDW and clinical outcomes in many human diseases. Several plausible explanations have been postulated to explain the relationship between RDW and adverse outcomes. First, a high RDW level indicates a great degree of heterogeneity in RBC size (anisocytosis). In addition, accelerating RBC destruction and/or ineffective erythropoiesis, which are common in patients undergoing dialysis, and bone marrow dysfunction can lead to a high RDW level. Bone marrow-derived mesenchymal stem cells have been reported to play a crucial role in the restoration of many injured vital organs^22^. Therefore, disordered hematopoiesis in dialysis patients may contribute to the high risk of mortality associated with a high RDW. Second, inflammation, which is prevalent in CKD patients, has been closely linked with RDW in many patient populations. Proinflammatory cytokines are well known to inhibit erythropoietin-induced RBC maturation and proliferation^23^. Solak *et al*. reported a strong association between RDW and CRP in predialysis CKD patients^13^. A similar relationship has also been found in patients undergoing PD and HD^19^,^20^. Furthermore, inflammation has been shown to be associated with mortality in dialysis patients. Third, the presence of malnutrition and/or protein energy wasting, which is common in dialysis patients, is known to increase RDW. RDW has also been reported to be significantly and inversely correlated with nutritional index in a wide array of medical conditions^12^,^24^. Fourth, distinct from the investigation by Peng *et al*., we found that residual renal function was greater in the low RDW group than that in the high RDW group. Residual renal function has been shown to have a beneficial effect on patient survival, and especially in patients undergoing PD^25^. In addition, the loss of residual renal function has been implicated in malnutrition and increased inflammation^26^.

Other potential mechanisms have also been proposed to explain the association between RDW and unfavorable outcomes. RDW has been associated with slow coronary flow and left ventricular filling pressure in patients with diastolic heart failure^27^,^28^. In patients with stage 1–5 CKD, RDW has been shown to independently predict endothelial dysfunction, which may be responsible for the high CVD burden in patients with CKD^13^. In addition, a recent study of patients with advanced CKD by Leszek *et al*. reported a relationship between RDW and left ventricular diastolic dysfunction^29^. Elevated RDW may lead to increased mortality through impaired microcirculation, ischemia and thrombosis as a result of reduced RBC deformability^30^. Finally, anisocytosis also enhances the accumulation of erythrocytes in the atherosclerotic lesions, leading to the neutralization of vasodilators, growth, ulceration and thrombosis of the fibrous cap^31^.

There are several limitations to this study. First, as a retrospective and observational study, we could not prove a causal relationship between RDW and mortality. Second, a single measurement of RDW may underestimate the true relationship with study outcomes. Third, a single-center investigation limits the extrapolation of the findings to the whole PD population. Fourth, the possibility of an epiphenomenon reflecting the complex interactions between RDW and other un-evaluated risk factors cannot completely be excluded. For example, inflammation, oxidative stress and poor nutrition usually accompany CKD and they are associated with impaired production of erythropoietin, which promotes the release of erythrocytes of heterogeneous size from the bone marrow^31^,^32^. However, oxidative stress was not evaluated in this study. Therefore, RDW should not be considered a causative factor, but rather a valuable marker in assessing the risk of mortality. Fifth, Lippi *et al*. reported the lack of harmonization of RDW by using four different hematological analyzers^33^. The optimal cut-off value of RDW for the prediction of mortality risk might vary and depend on the used hemocytometer.

In conclusion, we found that a higher RDW was an independent risk factor for overall and CVD-related mortality in patients undergoing CAPD, and that its predictive value was better than other makers of anemia. It is unclear whether RDW is a true risk factor for mortality or merely an integrated biomarker reflecting anemia, inflammation, malnutrition and impaired kidney function. Nevertheless, given that RDW is a readily available hematological parameter without the need for additional cost, we suggest that it can be used as a valuable index to stratify the risk of mortality beyond a diagnosis of anemia. Further investigations are required to verify our findings and elucidate the underlying mechanisms.

## Materials and Methods

This retrospective longitudinal study was performed at a single PD center at Changhua Christian Hospital, Taiwan. We recruited all incident patients who started CAPD as renal replacement therapy between 1 January 2006 and 31 October 2014. Patients were excluded if they were under 18 years of age (n = 5) and had received PD for less than 3 months (n = 8). The final study cohort consisted of 313 adult patients undergoing CAPD, all of whom were followed from the index date, defined as the date of initiating CAPD, until the date of death or the end of the study period (31 October 2015), whichever occurred first.

Baseline data on socio-demographics, body mass index (BMI), pre-dialysis status (pre-dialysis CKD, failed transplant or hemodialysis), smoking status, the underlying cause of CKD, comorbidities, laboratory variables, medications used and PD-related parameters were obtained from our PD database and review of medical records and used for statistical analysis. The comorbidities included diabetes mellitus, hypertension, cancer, dementia, chronic lung disease, liver cirrhosis, hyperlipidemia and cardiovascular disease (CVD). Laboratory variables included blood levels of BUN, creatinine, albumin, glutamic-pyruvic transaminase (GPT), white blood cell (WBC) count, alkaline phosphate (ALP), hemoglobin, RDW, ferritin, transferrin saturation, cholesterol, triglyceride, intact parathyroid hormone (PTH), calcium, and phosphate. The medications included angiotensin-converting enzyme (ACE) inhibitors, angiotensin II receptor blockers (ARB), anti-anemia agents (iron preparation, folic acid, and vitamin B12), erythropoiesis stimulating agents (ESA), and calcium supplement. PD-related parameters included weekly total Kt/V urea, nPNA, D/P (creatinine) at 4 hours, ultrafiltration, 24-hour urine output, and residual renal function.

Measurement of red blood cell parameters was carried out using the automatic hematology analyzer (DxH 800, Beckman Coulter). RDW was calculated using standardized methods and routinely reported as part of the complete blood cell count as a percentage. The whole study cohort was divided into two groups by the median RDW value (15.3%) to assess the predictive value of RDW on study outcomes as: the high RDW group (>15.3%), and low RDW group (≦15.3%). CVD was the leading cause of mortality, followed by infection. The study outcomes were all-cause and CVD-related mortality. This study was approved by the institutional review board of Changhua Christian Hospital and conducted in compliance with the declaration of Helsinki. Written informed consent was not required for this retrospective study due to its non-intrusiveness and patient anonymity.

### Statistical analysis

Descriptive data were expressed as number (N) and proportion, or mean ± standard deviation (SD) for categorical and continuous data, respectively. Differences between patients with high and those low RDW were compared using the Student’s test or Mann-Whitney test for continuous variables, and the chi-square test or Fisher’s exact test for categorical variables. Survival curves were calculated using the Kaplan-Meier method, and differences in survival were assessed using the log-rank test. Cox proportional hazard analysis was used to evaluate the association between RDW and study outcomes, including all-cause and CVD-related mortality, initially without adjustments. Multivariate Cox regression analysis was then performed with adjustments for the covariates which showed a significant correlation (p < 0.05) with the outcome of interest, including sex, age, BMI, smoking status, comorbidities, laboratory variables, medications and PD-related parameters.

Five models were used: model 1, adjusted for sex, age, smoking status and BMI; model 2, adjusted for all variables in model 1, plus medications; model 3, adjusted for all variables in model 2, plus comorbidities; model 4, adjusted for all variables in model 3, plus PD-related parameters; model 5, adjusted for all variables in model 4, plus laboratory data. Three sensitivity analyses were performed to increase the robustness of our results. First, the hazard ratio (HR) of RDW was calculated per 1% increment in RDW level to maximize the predictive value of RDW for the clinical outcomes of interest. Second, the entire cohort was divided into three tertiles by the RDW level with the first tertile group as the reference in relation to the risk of mortality. Third, the optimal RDW value determined from the ROC analysis was used to divide the patients for statistical analysis.

The associations between RDW and laboratory variables were tested using Pearson rank correlation test, and linear regression was used to determine the expected changes in RDW with each unit change in these continuous variables.

ROC analysis with AUC analysis was conducted to compare the predictive value of RDW for all-cause and CVD-related mortality within 1 year, 3 years and 5 years of initiating PD with other markers of anemia (hemoglobin, ferritin and iron saturation), albumin and WBC. The predictive value of these variables was further examined by the AUC with each variable added to those variables in Cox regression model 4. We also tested the possible association between RDW and mortality in subgroups of patients stratified by age, comorbid diseases (diabetes mellitus, hyperlipidemia, CVD), and medications (ACE inhibitors/ARB). A two-tailed p value of less than 0.05 was considered to be statistically significant, and all statistical analyses were performed using IBM SPSS Statistics for Windows, Version 22.0 (IBM Corp., Armonk, NY).

## Additional Information

**How to cite this article**: Hsieh, Y.-P. *et al*. Association between red cell distribution width and mortality in patients undergoing continuous ambulatory peritoneal dialysis. *Sci. Rep.* **7**, 45632; doi: 10.1038/srep45632 (2017).

**Publisher's note:** Springer Nature remains neutral with regard to jurisdictional claims in published maps and institutional affiliations.

## Figures and Tables

**Table 1:** Baseline characteristics of the study population by the RDW groups.

	RDW ≤ 15.3%	RDW > 15.3%	p-value
Number of patient	155	158	
Sex, men	86 (55.48%)	78 (49.37%)	0.332
Age (years)	51.21 ± 14.83	57.89 ± 16.45	<0.001*
Body mass index (kg/m^2^)	23.91 ± 3.77	23.55 ± 4.08	0.418
Smoker			0.763
never	125 (80.65%)	124 (78.48%)	
current	5 (3.23%)	4 (2.53%)	
ever	25 (16.13%)	30 (18.99%)	
Medication prescription			
ACE inhibitor/ARB	105 (67.74%)	106 (67.09%)	0.998
Erythropoiesis stimulating agents	146 (94.19%)	153 (96.84%)	0.287
Calcium supplement	141 (90.97%)	140 (88.61%)	0.577
Iron preparation	135 (87.1%)	123 (77.85%)	0.038*
Folic acid	149 (96.13%)	150 (94.94%)	0.786
Vitamin B12	5 (3.23%)	8 (5.06%)	0.573
Comorbidity			
Hypertension	150 (96.77%)	154 (97.47%)	0.748
Diabetes mellitus	60 (38.71%)	58 (36.71%)	0.804
Cardiovascular disease	55 (35.48%)	72 (45.57%)	0.089
Chronic lung disease	16 (10.32%)	28 (17.72%)	0.073
Liver cirrhosis	3 (1.94%)	4 (2.53%)	1.000
Dementia	1 (0.65%)	4 (2.53%)	0.371
Cancer	7 (4.52%)	11 (6.96%)	0.468
Hyperlipidemia	39 (25.16%)	48 (30.38%)	0.366
PD related parameters			
D/P Creatinine at 4 hours	0.68 ± 0.12	0.68 ± 0.13	0.708
Ultrafiltration (L/day)	0.74 ± 0.06	0.86 ± 0.14	0.369
24 hrs urine volume (L)	1.11 ± 0.65	0.85 ± 0.58	<0.001*
Total Weekly Kt/V	2.05 ± 0.51	1.95 ± 0.51	0.110
nPNA (g/kg/day)	0.98 ± 0.24	0.98 ± 0.28	0.940
Residual renal function (mL/min/1.73 m^2^)	3.42 ± 1.98	2.74 ± 1.87	0.002*
Laboratory data			
RDW (%)	14.37 ± 0.67	16.95 ± 1.74	<0.001*
Serum albumin (g/L)	34.1 ± 5.1	31.2 ± 5.7	<0.001*
ALP (μkat/L)	1.54 ± 1.03	1.85 ± 1.57	0.039*
BUN (mmol/L)	28.20 ± 7.37	30.21 ± 8.25	0.024*
Creatinine (μmol/L)	885.77 ± 240.45	843.34 ± 251.05	0.124
Cholesterol (mmol/L)	4.92 ± 1.23	4.62 ± 1.20	0.028*
Triglyceride (mmol/L)	1.55 ± 0.77	1.39 ± 0.69	0.064
Ferritin (ng/mL)	297.43 ± 240.13	390.61 ± 406	0.014*
GPT (μkat/L)	0.33 ± 0.31	0.36 ± 0.30	0.441
Hemoglobin (g/L)	90.1 ± 13.6	84.8 ± 10.9	<0.001*
Intact PTH (pmol/L)	43.81 ± 36.68	42.57 ± 31.98	0.750
Calcium (mmol/L)	2.12 ± 0.17	2.06 ± 0.19	0.006*
Phosphorus (mmol/L)	1.74 ± 0.37	1.76 ± 0.45	0.756
Transferrin Saturation (%)	0.28 ± 0.13	0.27 ± 0.15	0.809
WBC count (X10^9/L)	7.32 ± 2.4	7.36 ± 2.33	0.892
Calcium phosphate product (mmol^2^/L^2^)	3.66 ± 0.83	3.55 ± 0.95	0.265

Values are expressed as mean ± SD or number (percentage).

Abbreviations: ACE inhibitor, angiotensin-converting enzyme inhibitor; ARB, angiotensin II receptor blocker; BMI, body mass index; GPT, glutamic-pyruvic transaminase; WBC, white blood cell count; PTH, parathyroid hormone; ALP, alkaline phosphate; BUN, blood urea nitrogen; RDW, red cell distribution width.

*p-value < 0.05.

**Table 2:** Univariate and multivariate Cox regression models of all-cause and CVD-related mortality for RDW groups.

	All-cause mortality		CVD mortality	
	Hazard ratio (95% CI)	p-value	Hazard ratio (95% CI)	p-value
RDW median				
unadjusted model	2.95 (1.88, 4.64)	<0.001	3.06 (1.64, 5.71)	<0.001
Model 1	2.23 (1.41, 3.54)	0.001	2.48 (1.32, 4.69)	0.005
Model 2	2.36 (1.48, 3.78)	<0.001	2.69 (1.41, 5.13)	0.003
Model 3	2.60 (1.59, 4.26)	<0.001	3.38 (1.72, 6.67)	<0.001
Model 4	2.82 (1.72, 4.64)	<0.001	3.60 (1.8, 7.22)	<0.001
Model 5	2.58 (1.31, 5.09)	0.006	3.48 (1.44, 8.43)	0.006
(B) sensitivity tests				
(i) RDW as a continuous variable	1.15 (1.00, 1.33)	0.047	1.22 (1.00, 1.48)	0.049
(ii) optimal RDW by ROC analysis	2.74 (1.56, 4.81)	<0.001	3.26 (1.44, 7.39)	0.004
(iii) RDW tertiles				
First tertile	1		1	
Second tertile	1.34 (0.62, 2.93)	0.457	2.03 (0.62, 6.62)	0.241
Third tertile	2.41 (1.07, 5.44)	0.034	5.21 (1.46, 18.64)	0.011
p-trend		0.023		0.008

Model 1: RDW, age, sex, BMI and smoking status.

Model 2: Model 1 plus medications (ACE inhibitors/ARB, iron preparation, folic acid, and vitamin B12 supplement, erythropoiesis stimulating agents, and calcium supplement).

Model 3: Model 2 plus comorbidities (diabetes mellitus, hypertension, cancer, dementia and chronic lung disease, liver cirrhosis, hyperlipidemia and cardiovascular disease).

Model 4: Model 3 plus PD related parameters (weekly total Kt/V urea, nPNA, D/P creatinine at 4 hours, ultrafiltration, 24-hour urine output, and residual renal function.).

Model 5: model 4 plus laboratory data (BUN, creatinine, albumin, GPT, WBC counts, alkaline phosphate, hemoglobin, ferritin, transferrin saturation, cholesterol, triglyceride, intact PTH, calcium, and phosphate).

**Table 3:** Linear regression coefficients and Pearson correlation of RDW with various laboratory data.

	Linear regression coefficients (95% CI)	Pearson correlation	p-value
Albumin (per 10-g/L increment)	−0.646 (−1.15, −0.14)	−0.196	0.013*
Ferritin (per 100-ng/mL increment)	0.026 (−0.04, 0.09)	0.047	0.458
WBC (per 10^9-cells/L increment)	−0.02 (−0.12, 0.08)	−0.026	0.682
Hemoglobin (per 10-g/L increment)	−0.368 (−0.54, −0.2)	−0.251	<0.001*
Iron saturation (per 1% increment)	−0.398 (−1.94, 1.14)	−0.030	0.613
iPTH (per 100- pmol/L increment)	−0.999 (−1.6965, −0.377)	−0.185	0.003*
BMI (per 1-kg/m^2^ increment)	−0.071 (−0.12, −0.02)	−0.151	0.009*
Creatinine (per 100-μmol/L increment)	−0.087 (−0.192, 0.011)	−0.117	0.087
Calcium (per 1-mmol/L increment)	−0.656 (−1.76, 0.44)	−0.066	0.244
ALP (per 1-μkat/L increment)	0 (−16.47, 16.47)	0.000	0.999
BUN (per 1-mmol/L increment)	1.896 (−1.064, 4.845)	0.081	0.209
Cholesterol (per 1-mmol/L increment)	−0.196 (−0.366, −0.027)	−0.130	0.024*
GPT (per 1-μkat/L increment)	0.235 (−38.82, 88.23)	0.041	0.447
Phosphorus (per 1-mmol/L increment)	0.482 (−0.185, 1.145)	0.108	0.156
Triglyceride (per 100-mmol/L increment)	−4.69 (−33.63, 23.89)	−0.019	0.749

Abbreviations: BMI, body mass index; GPT, glutamic-pyruvic transaminase; WBC, white blood cell count; PTH, parathyroid hormone; ALP, alkaline phosphate; BUN, blood urea nitrogen; RDW, red cell distribution width.

*p < 0.05.

**Table 4:** AUC using ROC curve analysis to predict all-cause and cardiovascular mortality by various parameters.

	All-cause mortality			Cardiovascular mortality		
	1-year	3-year	5-year	1-year	3-year	5-year
AUC for each variable						
RDW	0.791	0.726	0.699	0.769	0.743	0.74
	(0.72, 0.87)	(0.66, 0.8)	(0.62, 0.78)	(0.72, 0.82)	(0.66, 0.83)	(0.66, 0.82)
Albumin	0.785	0.757	0.683	0.748	0.717	0.673
	(0.7, 0.87)	(0.69, 0.83)	(0.6, 0.76)	(0.59, 0.9)	(0.62, 0.82)	(0.57, 0.78)
Hemoglobin	0.580	0.528	0.51	0.504	0.48	0.475
	(0.46, 0.7)	(0.45, 0.61)	(0.43, 0.59)	(0.35, 0.65)	(0.38, 0.58)	(0.37, 0.58)
Ferritin	0.684	0.599	0.584	0.602	0.568	0.538
	(0.58, 0.79)	(0.52, 0.67)	(0.51, 0.65)	(0.47, 0.74)	(0.47, 0.66)	(0.44, 0.63)
Iron saturation	0.63	0.538	0.535	0.597	0.487	0.491
	(0.51, 0.75)	(0.45, 0.62)	(0.46, 0.61)	(0.45,0.74)	(0.37, 0.6)	(0.39, 0.59)
White blood cell count	0.735	0.58	0.588	0.583	0.506	0.492
	(0.62, 0.85)	(0.49, 0.67)	(0.51, 0.66)	(0.42, 0.75)	(0.39, 0.62)	(0.39, 0.6)
AUC for variables in model 4 plus the following variable						
Variables in model 4 + RDW	0.916	0.892	0.876	0.924	0.875	0.882
	(0.88, 0.96)	(0.85, 0.93)	(0.83, 0.92)	(0.86, 0.99)	(0.81, 0.94)	(0.83, 0.94)
Variables in model 4 + albumin	0.905	0.876	0.866	0.899	0.829	0.843
	(0.86, 0.95)	(0.83, 0.92)	(0.82, 0.92)	(0.83, 0.97)	(0.76, 0.9)	(0.78, 0.91)
Variables in model 4 + hemoglobin	0.902	0.867	0.847	0.816	0.822	0.831
	(0.86, 0.94)	(0.82, 0.91)	(0.79,0.9)	(0.72, 0.91)	(0.76, 0.88)	(0.78, 0.89)
Variables in model 4 + ferritin	0.911	0.877	0.854	0.862	0.824	0.831
	(0.86, 0.96)	(0.84, 0.92)	(0.8, 0.91)	(0.79,0.94)	(0.76, 0.89)	(0.77, 0.9)
Variables in model 4 + iron saturation	0.889	0.888	0.875	0.914	0.849	0.855
	(0.84, 0.94)	(0.85, 0.93)	(0.83, 0.92)	(0.85, 0.98)	(0.78, 0.92)	(0.8, 0.91)
Variables in model 4 + White blood cell count	0.895	0.88	0.872	0.896	0.836	0.847
	(0.84, 0.94)	(0.84, 0.92)	(0.82, 0.92)	(0.81, 0.98)	(0.77, 0.9)	(0.79, 0.9)

Data are presented as AUC (95% confidence interval).

Abbreviations: AUC, area under the curve; ROC, receiver operating characteristic.

Variables in model 4 included age, sex, body mass index, smoking status, medications usage, comorbid conditions and peritoneal dialysis related parameters.
